# Adiponectin: a manifold therapeutic target for metabolic syndrome, diabetes, and coronary disease?

**DOI:** 10.1186/1475-2840-13-103

**Published:** 2014-06-23

**Authors:** Enrique Z Fisman, Alexander Tenenbaum

**Affiliations:** 1 Sackler Faculty of Medicine, Tel Aviv University, Tel Aviv 69978, Israel; 2 Cardiovascular Diabetology Research Foundation, Holon 58484, Israel; 3 Cardiac Rehabilitation Institute, Sheba Medical Center, Tel Hashomer 52621, Israel

**Keywords:** Adipokines, Adiponectin, Atherosclerosis, Coronary artery disease, Diabetes mellitus, Metabolic syndrome, Obesity, T-cadherin

## Abstract

Adiponectin is the most abundant peptide secreted by adipocytes, being a key component in the interrelationship between adiposity, insulin resistance and inflammation. Central obesity accompanied by insulin resistance is a key factor in the development of metabolic syndrome (MS) and future macrovascular complications. Moreover, the remarkable correlation between coronary artery disease (CAD) and alterations in glucose metabolism has raised the likelihood that atherosclerosis and type 2 diabetes mellitus (T2DM) may share a common biological background. We summarize here the current knowledge about the influence of adiponectin on insulin sensitivity and endothelial function, discussing its forthcoming prospects and potential role as a therapeutic target for MS, T2DM, and cardiovascular disease. Adiponectin is present in the circulation as a dimer, trimer or protein complex of high molecular weight hexamers, >400 kDa. AdipoR1 and AdipoR2 are its major receptors in vivo mediating the metabolic actions. Adiponectin stimulates phosphorylation and AMP (adenosin mono phosphate) kinase activation, exerting direct effects on vascular endothelium, diminishing the inflammatory response to mechanical injury and enhancing endothelium protection in cases of apolipoprotein E deficiency. Hypoadiponectinemia is consistently associated with obesity, MS, atherosclerosis, CAD, T2DM. Lifestyle correction helps to favorably modify plasma adiponectin levels. Low adiponectinemia in obese patients is raised via continued weight loss programs in both diabetic and nondiabetic individuals and is also accompanied by reductions in pro-inflammatory factors. Diet modifications, like intake of fish, omega-3 supplementation, adherence to a Mediterranean dietary pattern and coffee consumption also increase adiponectin levels. Antidiabetic and cardiovascular pharmacological agents, like glitazones, glimepiride, angiotensin converting enzyme inhibitors and angiotensin receptor blockers are also able to improve adiponectin concentration. Fibric acid derivatives, like bezafibrate and fenofibrate, have been reported to enhance adiponectin levels as well. T-cadherin, a membrane-associated adiponectin-binding protein lacking intracellular domain seems to be a main mediator of the antiatherogenic adiponectin actions. The finding of novel pharmacologic agents proficient to improve adiponectin plasma levels should be target of exhaustive research. Interesting future approaches could be the development of adiponectin-targeted drugs chemically designed to induce the activaton of its receptors and/or postreceptor signaling pathways, or the development of specific adiponectin agonists.

## Background

The classical view of adipose tissue as just a passive reservoir for energy storage has radically changed. Two types of adipose tissue are found in mammals, brown and white, each of them with different physiological roles. Brown adipose tissue has specialized functions in thermogenesis through oxidation of fatty acids due to the presence of its specific uncoupling protein (UCP1), which uncouples thermogenic oxidative phosphorylation [1]. Instead, white adipose tissue stores energy in the form of triglycerides and, in situations of energy deficit such as fasting, supplies fatty acids to the circulation.

Thus, white adipose tissue is nowadays perceived as an important organ involved in energy homeostasis and body weight control. Besides its function as an energy reservoir, it plays a key role as an organ secreting numerous bioactive molecules collectively called adipokines or adipocytokines [2]; the first term will be used along the present review. The number of identified adipokines is permanently increasing, as well as their potential clinical diagnostic and prognostic value. These adipokines include mainly adiponectin [2-5], leptin [5], tumor necrosis factor (TNF) alpha [6,7], osteoprotegerin [8] interleukin 6 (IL-6) [9], resistin [10], interleukin 1 (IL-1) [11,12], apelin [13], visfatin [14], monocyte chemotactic protein-1 (MCP-1) [15,16], plasminogen activator inhibitor-1 (PAI-1) [17], retinol binding protein 4 (RBP4) [18] and several others.

The adipokines are involved in the regulation of body fat accumulation, adipose tissue development, energy metabolism and control of food intake, and play also a dominant role in the pathophysiology of several metabolic disorders [2-6]. Namely, an abnormal regulation in adipokines production will facilitate a biochemical imbalance potentially leading to the development of various ailments and diseases, mainly obesity, insulin resistance (IR) and atherosclerosis, among others [2,10,19]. It should be pinpointed that not all fatty deposits behave according to the same pathophysiological pattern [20,21]. In particular, it has been shown that visceral fat deposits are more metabolically active than their subcutaneous homologues, being particularly involved in the development of diseases associated with obesity, such as the metabolic syndrome (MS), type 2 diabetes mellitus (T2DM) and coronary artery disease (CAD) [21].

Adiponectin is the most abundant peptide secreted by adipocytes [3,22], being a key component in the interrelationship between adiposity, insulin resistance and inflammation [22]. Central obesity accompanied by insulin resistance is a key factor in the development of MS and future macrovascular complications [23]. Moreover, the remarkable correlation between CAD and alterations in glucose metabolism has raised the likelihood that atherosclerosis and T2DM may share a common biological background [24,25]. Large-vessel atherosclerosis can precede the development of diabetes, suggesting that rather than atherosclerosis being a complication of diabetes, both conditions may share similar genetic and acquired characteristics, a "common soil" [26].

In the present review we summarize the current knowledge about the influence of adiponectin on insulin sensitivity and endothelial function, discussing its forthcoming prospects and potential role as a manifold therapeutic target for MS, diabetes, and cardiovascular disease.

### Genetics, structure and circulating levels

Several studies have revealed a moderate to high estimate of heritability (30–70%) for plasma adiponectin levels, which are influenced by the interplay of several genes [27-30]. A meta-analysis of genome-wide association studies performed in nearly 40000 individuals in order to identify genes associated with adiponectin levels, revealed 8 loci and confirmed other 2 previously reported loci [31]. One of the main loci seems to be on chromosome 3q27, which contains a susceptibility locus for T2DM and MS [32]. Reduced adiponectin levels can be caused by genetic factors, such as the single nucleotide polymorphism (SNP) 276 in the adiponectin gene itself [33].

Analyses of SNP and mutations in the adiponectin gene have suggested a relationship between adiponectin and glucose metabolism diseases. For instance, SNP at position 94 associates closely with T2DM, as do SNP45 and SNP276 [34,35], and SNP rs266729 was found to be significantly associated with higher odds of CAD [33]. Unfavorable effects of the AdipoQ 45 T/G SNP on lipid profile and glucose metabolism have also been described [36]. Moreover, the latter polymorphism is also strongly correlated with CAD in T2DM subjects [37]. Interestingly, it has been suggested that primary genetic lesions that lower adiponectin levels may result in hypertension [38]; decreased circulating adiponectin and hypertension correlated significantly with the I164T polymorphism [39].

Adiponectin is a protein consisting of 244 aminoacids displaying structural similarities to collagen and TNF-alpha, and is mostly located in adipocytes. Adiponectin was independently identified by several research groups using different techniques, receiving different names like ACRP30, AdipoQ and apM1 [40-42]. Adiponectin is a protein of 30 kDa present in the circulation as a dimer, trimer or as a protein complex of high molecular weight (HMW) hexamers, >400 kDa, in which the oligomers control the biological activity of the protein [43]. The higher order structures include also low-molecular weight (LMW) hexamers of 180 kDa. Adiponectin can exist in plasma in its complete form or in globular fragments; the first appears to be the most common form. It circulates at physiological concentrations that represent about 0.05% of all plasma proteins [22].

The normal circulating values were initially set at 5–30 μg/ml [42], albeit subsequent investigations reported a much narrower range - 5–10 μg/ml – [44,45]. It should be pinpointed that ethnic and gender differences are present; values are higher in Caucasians than in Indo-Asians [46] and in women than in men [44], albeit significant gender differences in adiponectin concentrations were not observed in a Sudanese population [47]. Significantly lower values have been reported in women with gestational diabetes [48] and during menopause [49]. Concentrations are at lower normal limits in obese subjects [44], and reduced in MS, both in humans [50] and in experimental animal models [51].

Importantly, adiponectin values are also systematically lower in diabetics compared to non-diabetics, no matter to what heart failure staging class they belong [52]. An exception to the general rule linking increased adiponectin levels with a better outcome seems to be non-ischemic cardiomyopathy, in which despite of its high peripheral concentrations, it does not show cardioprotective effects [53].

### General bioactivity

Mice studies have confirmed that adiponectin receptors AdipoR1 and AdipoR2 are its major receptors in vivo [54,55] mediating the metabolic actions. These effects are also dependent on specific tissues, with muscular AdipoR1 involved in stimulating adenosin mono phosphate (AMP) activated protein kinase, while hepatic AdipoR2 is involved mainly in activation of the peroxisome proliferator activated receptor (PPAR) gamma. Both AdipoR1 and AdipoR2 serve as receptors for globular and full-length adiponectin and mediate also increased fatty-acid oxidation and glucose uptake [55,56].

Adiponectin, both in its globular and HMW forms, stimulates phosphorylation and AMP kinase activation in skeletal muscle. Anyway, the use of full-length adiponectin produced by mammalian cells suggests that the liver and not muscle is the primary site of adiponectin bioactivity [57]. In addition to AMP kinase activation, adiponectin induces carboxylase acetyl-coenzyme A phosphorylation, glucose uptake, nitric oxide synthesis, lactate production in myocytes, and reduced liver production of molecules involved in gluconeogenesis. These effects seem responsible for the lowering of glucose levels in vivo, via glucose utilization and fatty-acid oxidation by activating AMP-activated protein kinase [58]. T-cadherin, a membrane-associated adiponectin-binding protein localized in vascular smooth muscle cells and endothelial cells, seems to be the mediator of adiponectin activity [59].

In a mammalian expression system, full-length adiponectin is produced and secreted both as LMW and HMW complexes. Interestingly, its administration to normal weight or obese-diabetic mice results in a decreased serum glucose [57,60]. The effect of adiponectin on the liver requires hydroxylation and glycosylation of residues within the collagenous domain of adiponectin [61]. This finding may explain why studies employing the globular form (lacking the collagenous domain) or the bacterially produced full-length form (lacking post-translational modifications in the collagenous domain) do not affect hepatic glucose metabolism or insulin sensitivity [60]. It has been shown that only HMW adiponectin decreases after a glucose load, suggesting that the HMW form of adiponectin is prone to be affected more rapidly than its LMW or medium–molecular weight counterparts. The mechanism remains unclear; possibly explanations may include decreased secretion of HMW adiponectin by adipocytes, augmented clearance of HMW adiponectin from the circulation, increased metabolism of HMW adiponectin, or a combination of these facts [62]. Adiponectin is very stable in vivo compared with other adipokines, since its half-life is very much longer, ranging from 2.5 [63] to 14 hours [64].

Another important point that should be mentioned is that serum adiponectin is inversely related to body fat mass and to the degree of insulin resistance. Its concentration is particularly low in adults with T2DM or CAD. It is accepted, therefore, that adiponectin ameliorates sensitivity to insulin and contributes to cardiovascular protection [65-67]. Low circulating levels, particularly of the HMW component [68,69], are also a strong risk marker for the development of the MS.

Hypoadiponectinemia is also associated with elevated intramyocellular and intrahepatic lipid content, as seen in non-alcoholic fatty liver and non-alcoholic steatohepatitis, additional indicators of dyslipidemia not currently included as risk factors for the MS [70-72], showing an inverse relationship with vascular endothelial growth factor levels in some inflammatory settings [73].

### Adiponectin and insulin sensitivity

Adiponectin increases the sensitivity to insulin through several mechanisms. AdipoR1 and AdipoR2 are transmembrane receptors, whose carboxyl terminal group (C-terminal) is located outside the membrane, and the amino terminal group (N-terminal) inside [74]. When adiponectin attaches to its receptor it activates AMP kinase [19,67], promoting so glucose uptake by muscles via intracellular translocation of the GLUT4 transporters. Simultaneously, it hampers gluconeogenesis by inhibiting the hepatic enzyme phosphoenolpyruvate carboxylase, inhibits the synthesis of fatty acids and stimulates their oxidation [22,67].

Independently, adiponectin acts as an agonist of the peroxisome proliferator activated receptor (PPAR) gamma leading to additional uptake of plasmatic glucose [67]. In this context, the adiponectin-resistin index provides a good indicator for an increased risk of future development of T2DM and MS [75]. Finally, adiponectin enhances insulin sensitivity by increasing hepatic insulin receptor substrate 2 (IRS-2) expression via a macrophage-derived IL-6-dependent pathway [66]. Thus, these multiple pathways confer to adiponectin a key role in ensuring an effective protection against the development of insulin resistance (IR).

### Adiponectin and endothelial function

It has been shown that adiponectin exerts direct effects on vascular endothelium, diminishing the inflammatory response to mechanical injury and enhancing endothelium protection in cases of apolipoprotein E deficiency [43,76,77]. Regarding other lipids, cross-sectional studies showed, after adjusting for gender and adiposity, that adiponectin levels present a inverse correlation with triglycerides [78], while they are directly correlated with HDL-cholesterol [79].

It has been found that adiponectin plasma concentrations are lower in individuals with CAD compared to age- and obesity-matched controls [80] and that individuals with adiponectin levels under 4 μg/ml were at increased risk of CAD and presented more factors for MS [81]. Conversely, while prospectively evaluating men without CAD, it was found after a 6 year-follow up that individuals in the highest percentile of plasma adiponectin were at a lower risk of MI, compared with those in the lowest percentile [82]. Adiponectin levels are also decreased in people with hypertension, regardless the presence of insulin resistance [83]. These subjects are characterized by a decreased endothelium-dependent vasodilation, which could be one of the mechanisms involved in central obesity-associated hypertension [84].

**Figure 1:**
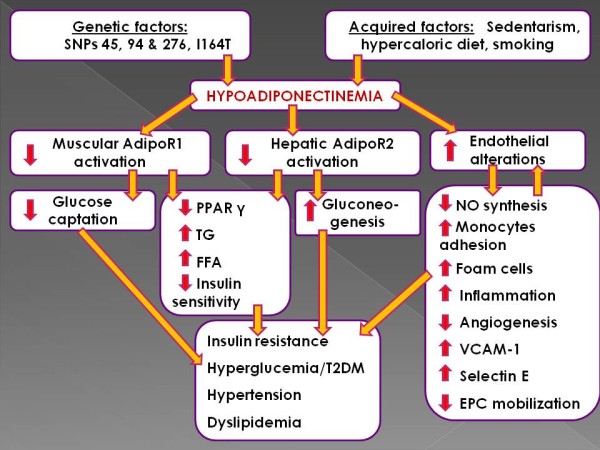
**Schematic depiction of the clinical outcomes of hypoadiponectinemia.** Hypoadiponectinemia leads to diminished adiponectin receptors activation accompanied by increased endothelial alterations. These factors put forth several biochemical chain reactions exerting detrimental consequences via multiple pathways. These chain reactions may act reciprocally, finally conducting to serious cardiometabolic derangement.

It is well established that adiponectin has an antiatherosclerotic effect via inhibition of adhesion molecules production, such as vascular cell adhesion protein 1 (VCAM-1) and selectin E [85,86]. The adiponectin-mediated suppression of nuclear factor kB, could be an important molecular mechanism for inhibiting monocytes adhesion to endothelial cells [86]. Immunohistochemistry studies show that adiponectin is not incorporated into the normal and intact vessel wall, while it presents a marked adherence to previously damaged vessel walls, like those mechanically injured by balloon catheters [87], and adiponectin may also act as a modulator for macrophage-to-foam cell transformation, slowing or inhibiting the process [88]. Moreover, experimental and clinical investigations indicate that adiponectin promotes endothelial repair and angiogenesis by increasing the number and function of endothelial progenitor cells (EPCs) [89-91]. This EPCs-mediated endothelial repair involves several stages, beginning with mobilization of EPCs from bone marrow or spleen into the bloodstream, followed by recruitment and adhesion of EPCs to the injured blood vessel wall, and finally, differentiation and tubule formation. Thus, adiponectin modulates almost every step of endothelial repair via EPCs [92,93]. A schematic representation of the multiple detrimental biological and clinical effects of hypoadiponectinemia is depicted in Figure 1.

### Current and forthcoming therapeutic prospects

Adiponectin levels may be negatively influenced by lifestyle, such as sedentarism, a high-fat diet causing obesity, or excessive smoking [94]. This influence can be reversed; lifestyle correction helps to favorably modify plasma adiponectin levels. Low adiponectinemia in obese patients was raised via continued weight loss programs in both diabetic and nondiabetic individuals [95,96], in obese adolescents [97] and was also accompanied by reductions in pro-inflammatory factors like IL-6, leptin and TNF alpha [98]. Moreover, increased adiponectin levels were already apparent after 1 week (two to three bouts) of moderately intense aerobic exercise, in some cases up to 260% [99]. Regarding diet modifications, several studies reported that daily intake of fish or omega-3 supplementation increased adiponectin levels by amounts ranging from 14 to 60% [100]. Furthermore, adherence to a Mediterranean dietary pattern showed excellent results in T2DM women [101]. Coffee consumption has also shown beneficial effects on adiponectin levels [102].

Unfortunately, adiponectin itself cannot be administered orally since its main component is a protein which is dissolved by the digestive system enzymes, being thus unable to reach the bloodstream. On the other hand, adiponectin levels may be pharmacologically modified. In this context, it has been found that antidiabetic treatment with either insulin or metformin in experimental models - albeit not able to improve adiponectin induced vasodilation and endothelial function - inhibits both the development of hypoadiponectinemia and the downregulation of the adaptor protein APPL1 in mesenteric resistance arteries [103]. Moreover, adiponectin concentrations increase after pioglitazone therapy in subjects with impaired glucose tolerance; glitazones also improve adiponectin levels in normal, obese, and T2DM subjects [104]. It should be highlighted that baseline adiponectin levels do not predict the response to glitazones [105]. Anyway, the use of glitazones to increase adiponectin is discouraged due to the potential adverse cardiovascular effects of these drugs, like heart failure or stroke [106], especially in the case of rosiglitazone [107]. The sulfonylurea glimepiride yields also positive effects on adiponectin, particularly in elderly T2DM patients [108].

Bezafibrate, a fibric acid derivative known for its capability to attenuate the progression of IR in CAD patients [109] and the declining of beta cells function in T2DM [110] has been reported to enhance adiponectin levels, partly acting through PPAR alpha stimulation [111]. Similar properties were reported for fenofibrate [111,112].

Cardiovascular drugs, as renin-angiotensin system blocking agents and angiotensin converting enzyme inhibitors significantly increase adiponectin levels and improve insulin sensitivity without affecting the degree of body adiposity [113-115]. For instance, telmisartan upregulates the expression of myocardial adiponectin, its receptor adipoR2, as well as GLUT4. Simultaneously, it also induces a protective role on the vascular system by upregulating the expression of adipoR1 and downregulating the expression of MCP-1 and nuclear factor kappa B (NF-κB) in the abdominal aorta in experimental animal models [116]. Co-administration of candesartan and pioglitazone during 6 months to hypertensive patients with T2DM significantly improved the baseline values of HMW adiponectin [117]. A potential mechanism for renin angiotensin system blocking agents to affect adiponectin levels seems to be promotion of adipogenic differentiation of preadipocytes [118] via PPAR gamma activity [119].

Attempts to increase adiponectin were also performed with nutraceutical agents like the herb derivatives astragaloside II and isoastragaloside, with satisfactory results in rodents [120,121]. In contrast, the use of purified allicin (the active ingredient in garlic) was unsuccessful [122].

With the increasing prevalence of T2DM and obesity, new technologies are developed to more easily monitor adiponectin levels or its potential surrogates. Currently, the concentration of total adiponectin maybe obtained by a using a commercially available human adiponectin radioimmunoassay kit [123] or enzyme-linked immunosorbent assays [124]. It has been shown that salivary pH is directly and significantly correlated to plasma adiponectin levels in premenopausal and menopausal women [125]. Should this condition be confirmed for other populations, salivary pH determination could represent an additional noninvasive, simple, and inexpensive surrogate for adiponectin assessment [126,127]. Urinary adiponectin can also be measured, and an increased concentration is associated with microalbuminuria and boteh micro- and macrovascular complications [128]. Anyway, laboratory methods for adiponectin measurement still require a more appropriate standardization, and this is also applicable to the determination of ideally therapeutic adiponectin levels for given clinical settings. It should be mentioned that excessively high concentrations may be undesirable; it has been reported that increased serum adiponectin and HOMA-IR could be associated with an augmented risk for the presence and development of cardiac autonomic neuropathy [129].

## Conclusions

As highlighted above, both functional and genetic studies on adiponectin strongly depict it as a key adipokine. Reduced adiponectin levels seem to be not just a mere biomarker of ailment, but play a causal role in the development of IR, MS, T2DM, hypertension, dyslipidemia and atherosclerosis [33,130]. On the other hand, favorable effects of a given adipokine on either diabetes or atherosclerosis predict similar effects on the other [131]. Hence, taking into consideration the high world prevalence of obesity, MS, T2DM and CAD, the possibility of a defined and unique therapeutic target to simultaneously combat their development becomes increasingly important [95].

Since adiponectin levels are consistently inversely correlated with each of these ailments, the finding of pharmacologic agents proficient to improve its plasma levels should be target of exhaustive research. An interesting approach could be the development of adiponectin-targeted drugs chemically designed to induce the activaton of its receptors and/or postreceptor signaling pathways. Such a move may also be able to reverse “adiponectin resistance”, which has been observed in both experimental and human research models [121,132]. Moreover, orally active AdipoR1 and AdipoR2 agonists were already satisfactorily used in rodent models [133]. T-cadherin, a membrane-associated adiponectin-binding protein lacking intracellular domain [134,135] seems to be a main mediator of the antiatherogenic adiponectin actions, and maybe a component of insulin granules [136]. Both adiponectin and T-cadherin were found to be inversely associated with human aortic and coronary atherosclerosis [59], and it appears that a majority of the whole body adiponectin is conveyed to cardiovascular tissues by T-cadherin [134,137,138]. T-cadherin seems to be a clue novel signaling pathway at the crossroads of vascular and metabolic disorders [139,140]. Hence, adiponectin represents in fact a multilayered therapeutic target for MS, diabetes and CAD. Investigating the intimate biochemical relationship between adiponectin, its receptors AdipoR1 and AdipoR2, and T-cadherin within the cardiovascular system could be a very promising avenue for the development of specific adiponectin agonists.

## Abbreviations

AMP: Adenosin mono phosphate; AMPK: Adenosine monophosphate-activated protein kinase; CAD: Coronary artery disease; EPCs: Endothelial progenitor cells; FFA: Free fatty acids; HMW: High molecular weight; IR: Insulin resistance; LMW: Low molecular weight; MCP-1: monocyte chemotactic protein-1; MS: metabolic syndrome; NO: Nitric oxide; PPAR: Peroxisome proliferator activated receptor; SNP: Single nucleotide polymorphism; TG: Triglycerides; TNF: Tumor necrosis factor; T2DM: Type 2 diabetes mellitus; VCAM-1: Vascular cell adhesion protein 1.

## Competing interests

The authors declare that they have no competing interests.

## Authors’ contributions

Enrique Z Fisman and Alexander Tenenbaum have equally contributed in the conception and drafting of the manuscript. Both authors read and approved the final manuscript.
